# Integrated mixed methods policy analysis for sustainable food systems: trends, challenges and future research

**DOI:** 10.1186/s40985-016-0040-5

**Published:** 2016-11-11

**Authors:** Soledad Cuevas

**Affiliations:** London School of Hygiene & Tropical Medicine / LCIRAH, London, UK

**Keywords:** Food policy, Food systems, Land use change, Palm oil, Mixed methods, Sustainability, Greenhouse gas emissions, Economics, Nutrition transition

## Abstract

Agriculture is a major contributor to greenhouse gas emissions, an important part of which is associated to deforestation and indirect land use change. Appropriate and coherent food policies can play an important role in aligning health, economic and environmental goals. From the point of view of policy analysis, however, this requires multi-sectoral, interdisciplinary approaches which can be highly complex. Important methodological advances in the area are not exempted from limitations and criticism.

We argue that there is scope for further developments in integrated quantitative and qualitative policy analysis combining existing methods, including mathematical modelling and stakeholder analysis. We outline methodological trends in the field, briefly characterise integrated mixed methods policy analysis and identify contributions, challenges and opportunities for future research. In particular, this type of approach can help address issues of uncertainty and context-specific validity, incorporate multiple perspectives and help advance meaningful interdisciplinary collaboration in the field. Substantial challenges remain, however, such as the integration of key issues related to non-communicable disease, or the incorporation of a broader range of qualitative approaches that can address important cultural and ethical dimensions of food.

## Background

Recent definitions of food sustainability have highlighted the existence of multiple inter-related dimensions including environmental, health, socioeconomic and cultural aspects [1]. Related to this shift towards a multi-dimensional concept of food sustainability, there has been increased emphasis on the understanding of food as a complex, integrated system [2]. This implies that environmental, health and other dimensions of sustainability need to be considered jointly, and the relevant interactions between them need to be accounted for.

In particular, certain topics such as the “food versus fuel” debate or the debate around the allocation of resources for animal feed versus plant-based food for direct human consumption have drawn attention to the importance of such interactions across sectors within the broader food system [3]. The most prominent examples are probably livestock or global flex crops [4] which have several food, energy and other industrial uses, such as palm oil and corn. In these sectors, complex environmental impacts, largely related to indirect land use change, interact with changes in global dietary patterns. For example, global increases in meat consumption as part of a wider process of “nutrition transition” have been associated to increases in non-communicable disease in high-income countries. At the same time, the use of land, water and other resources for animal feed has environmental impacts and can also push up prices of cereals and other non-animal food products, aggravating malnutrition, especially in low- and middle-income countries.

## Methodological trends and developments

On the one hand, the need to incorporate this complexity has led to significant methodological developments. These include the design and application of integrated conceptual frameworks [1], as well as complex multi-sector models [5–7]. A related trend has been the shift from traditional, attributional life-cycle analysis (LCA) towards a consequential, policy-focused LCA [3]. Consequential LCA attempts to include all of the relevant impacts of a certain policy across different sectors within the system, taking into account potential interactions [8].

Concerns have been raised, however, about the limitations of these increasingly complex models. In particular, researchers have pointed out the excessive uncertainty in the results as well as the lack of comparability in terms of both results, assumptions and methodologies [3]. In addition, important differences in language and approach can hamper interdisciplinary work in the area (ibid.). Finally, there has been increasing acknowledgement that realistic policy analysis requires an assessment not only of multiple objectives but also of the different and potentially conflictive perspectives of relevant actors [9]. Nevertheless, these issues are still comparatively neglected and analysis often focuses on policy options that are unrealistic given the specific context for which they are recommended.

On the other hand, approaches based on stakeholder analysis have frequently been applied to the fields of natural resource management, alongside land use planning and social forestry [10, 11] and, more rarely, sustainable diets and food systems [12]. Environmental Impact Assessments (EIA) also routinely incorporate stakeholder analysis, albeit generally from a very site-specific and geographically constrained perspective [13].

Stakeholder analysis is inherently context-specific although not necessarily bound by specific geographical or sectoral constraints. Moreover, the theoretical frameworks underlying these research methods, unlike most quantitative analysis in this field, tend to highlight the socially constructed nature of reality and focus explicitly on perspective and the existence of potentially conflicting objectives. Although this type of approach has its own set of limitations [14], it has been identified as being complementary to commonly used quantitative methods for research on sustainable food and therefore recommended for its use as part of mixed methods approaches.

What we mean by “integrated mixed-methods policy analysis for sustainable food systems” is a combination of quantitative economic and biophysical modelling and stakeholder analysis (or other qualitative methodologies) which aims to include different dimensions of sustainability across several sectors and their interactions, adopting a system perspective and a policy focus rather than addressing a specific site or technology. Similar methodological approaches have been recommended and applied in areas related to sustainable food systems over the last decade. In particular, variants of this type of approach have been recommended in fields such as sustainable nutrition at the household level [15], sustainable cropping [9], biofuels and food security [16] or biomass energy [13]. These methodologies are often used together with decision-making or “decision aiding” tools such as EIA, Multi-Criteria Decision Analysis (MCDA) or Back-casting.

The main contribution of this kind of approach is probably the explicit acknowledgement of different perspectives and possibly conflicting interests alongside the analysis of intersectoral impacts and linkages, increasing transparency and diversity in policy processes. Although this methodology can also itself be captured and manipulated by specific interests, it has frequently been applied to empower fringe, marginal or vulnerable stakeholders, and methods have been developed for this purpose, such as radical transactiveness [14]. In the case of food, these stakeholders can include smallholder farmers, workers in various segments of the industry or street food vendors, and vulnerable or low-income consumer groups, as well as more abstract entities, such as biodiversity. However, there are other relevant advantages which have been identified or suggested in the literature. Firstly, the use of methodologies that can combine quantitative and qualitative information can help to realistically manage uncertainty, dealing with different types of knowledge and uncertainty that are incorporated in food sustainability models, although often not explicitly recognised [3]. In addition, integrated methodologies can highlight the trade-off between context-specific validity and comparability, achieving a realistic balance and focusing the analysis on context-sensitive policy options [13]. Finally, mixed methods approaches can improve interdisciplinary collaboration, not by attempting to homogenise assumptions but rather by increasing the transparency and understanding of the differences in underlying theoretical frameworks across disciplines.

Despite the many opportunities offered by integrated mixed methods policy analysis, there remain significant challenges for its application to the field of sustainable food systems. Firstly, further work is needed in order to incorporate complex health and nutrition impacts. In particular, there is a need for further integration of emerging issues of non-communicable disease, where changing food environments and food processing mediate between health outcomes and environmental or socioeconomic impacts. Furthermore, the cultural and ethical aspects of diets are also frequently neglected in food policy analysis, despite being increasingly recognised as an integral dimension of sustainability. The adequate assessment of cultural and ethical implications of food policy might require broadening the range of qualitative methodologies within multi-sectoral policy analysis, including anthropological approaches at the household, industry and food environment levels [17]. To conclude, we argue that there is a need for further development of integrated mixed methods policy analysis to assess food sustainability, particularly on topics such as food biofuels, flex crops or livestock, which involve both indirect land use change and complex transformations in food environments and dietary patterns.

## Abbreviations

LCA: Life-cycle analysis
